# Functionalized electrospun nanofibers for high efficiency removal of particulate matter

**DOI:** 10.1038/s41598-022-12505-w

**Published:** 2022-05-19

**Authors:** Rethinam Senthil, Vijayan Sumathi, Alagumuthu Tamilselvi, Serdar Batıkan Kavukcu, A. Wilson Aruni

**Affiliations:** 1 Engineering Faculty, Leather Engineering Department, Ege University, 35100 Bornova, Izmir, Turkey; 2 School of Bio & Chemical Engineering, Sathyabama University, Chennai, Tamilnadu 600 199 India; 3 School of Electrical Engineering and Centre for Automation, VIT Chennai Campus, Chennai, Tamilnadu 600 127 India; 4 Unit for Science Dissemination, Central Leather Research Institute-CSIR, Chennai, 600020 India; 5 Science Faculty, Chemistry Department, Ege University, 35100 Bornova, Izmir, Turkey; 6 California University of Science and Medicine, 217 E Club Centre Dr suite a, San Bernardino, CA 92408 USA

**Keywords:** Environmental sciences, Nanoscience and technology

## Abstract

In recent years, introducing electrospun airfilters to enhance the removal of PM_2.5_ and PM_10–2.5_ has received much interest. In this study, a novel poly-(vinyl) alcohol (PVA)/carbon nanoparticle (CNP)/tea leaf extract (TLE), functionalized nanofibrous air filter (FNA) was fabricated using an electrospinning method. Novelty of the unique work in the blending of CNP and TLE, first of its kind, for the preparation of FNA. Polysaccharide crosslinked FNA has a carbon complex with two monosaccharide units to produce the intrinsic properties of the PM_2.5_ and PM_10–2.5_ removal efficiency. The FNA had promising traits of UV protection. The prepared FNA was characterized using physicochemical, mechanical, antimicrobial activity, etc., in addition to its PM_2.5_ and PM_10–2.5_ removal efficiency. Pore size and distribution study using the capillary flow porometry method has proved the structure of FNA. FNA exhibited excellent low pressure drop (110 Pa), which are promising characteristics for air purification. FNA from PVA: CNP: TLE exhibited high PM_2.5_ and PM_10–2.5_ removal efficiencies of 99.25% and 99.29%, respectively. Hence, the study proved.

## Introduction

Electrospinning is a unique technique for manufacturing nanostructured fibers with diameters ranging from the micro to the nano level. Polymeric-based electrospun materials with an average particle size of nanometers are extensively employed for airfilter applications. The materials are biocompatible, washable, reusable and low in weight. Micro and nanotechnology have benefited society in a variety of ways during the last few decades^1^. Nanofiber materials have attracted public attention due to their excellent surface fraction volume and prospective application in air filtration^2^.

Hazardous particulate matter (HPM) pollutants, which include particles, heavy metal dusts, toxic gases, spores, bacteria and organic pollutants including aerosol particles, benzene and polycyclic aromatic hydrocarbons are found in the atmosphere^3^. Small particles with a diameter of less than 2.5 µm in particular, run the risk of impairing human lung breathing and inhaling these particles raises the risk of teratogenic, carcinogenic and mutagenic effects as well as lung disorders such asthma, heart disease, stroke and lung cancer^4^. Effective protection of skin from exposure to sunlight needs a protective barrier so as to absorb or reflect the UV radiation before it reaches the skin surface^5^. Since, the attempt have been made to improve airfilters in order to improve their UV radiation protection efficiency.

Particular matter _2.5_ pollution particles in the air are made up of organic material such as elemental carbon and organic carbon, as well as inorganic matter such as SO^2^_4_, NO_3_^–^ and SiO_2_. Hazardous air pollution arises from a range of sources including biomass burning, industrial emissions, soil dust, aerosols and coal combustion^6^. The behaviour of PM particles is influenced by chemical compositions, morphologies and mechanical qualities^7^. The Capture of various air pollutants, an effective electrospun air filter is a preferable option. The effectiveness of an air filter is determined by the type of air pollution and the pollutant capture mechanism can be adjusted^8^. Electrospun based ultralow particulate air filters and high-efficiency particulate air filters capture small particulate matter with filtration effectiveness of 98.99 (%) and 98.98 (%), respectively^9^. The metal adsorption process is classified as chemisorption or physisorption. Electrostatic attraction can also produce. The interaction of phenolic compounds with the activated carbon surface occurred in a monolayer adsorption type, limited by their more metal adsorption abilities^10^.

Tea leaf is used as a natural low-cost for the adsorption of hazardous metal impurities to the existence of hydroxyl and carboxyl functional groups. Tea leaves waste is generated from cafeterias that could be used as an adsorbent to remove heavy metals from aqueous solutions. Tea waste is considered a viable choice due to its greater availability, low cost, and its efficiency in adsorbing hazardous ions such as Cr(VI), Ni, Pb, Cu, etc^11^. Removal of hazardous metals ions from debris air using agro wastes is evolving as a cost-effective solution to overcome the drawbacks of the existing method using costly chemicals^12^. Poly (vinyl) alcohol (PVA) is a biodegradable and biocompatible film-forming hydroxyl polymer with chemical stability and superior flexibility used in the preparation of functional membrane materials^13^. Nano filter membrane prepared using the blend of CNPs, TLE and PVA possessed improved PM removal efficiency.

Carbon nanoparticles have a wide range of features that may be interesting targets for a range of applications and their solubility or high stability in a variety of common solvents has led to the development of particular electrospun air filter applications^14^. Covalent attachment of polar functional groups such as NH_2_, OH, COH and COOH to the surface of carbon nanoparticles, as well as noncovalent adsorption of various functionalized molecules onto their surface area, gives carbon nanoparticles strength in polymeric solutions^15^. Poly (vinyl) alcohol has been shown to have good mechanical characteristics as one of the most common hydrophilic polymers. The poor metal adsorbent of PVA, on the other hand, is one of the most important barriers to its use. In light of this issue, solutions for modification have been explored. These characteristics make electrospun scaffolds a good replacement for airfilter applications^16^. Furthermore, combining antimicrobial CNPs and TLE with PVA-based FNA improves their antibacterial activity as well as metal adsorption of airborne particles.

In this study, FNA was developed to remove PM from polluted air. TLE and CNP, have good antibacterial properties against microorganisms and metal adsorption from contaminated air. We measured the various physical features of the produced fibers, such as shape, fiber size distribution and thermal stability for the application of antimicrobial airfiltration using PVA, CNPs and TLE.

## Materials and method

Olive oil was purchased from a local supermarket in Izmir, Turkey. Poly (vinyl) alcohol and deionised water were obtained from Sigma Aldrich, Turkey. Green tea leaf (*Camellia sinensis*) were obtained from Faculty of Agriculture, Ege University, Izmir, Turkey. Permission and proper guidance was done by Dean, Prof. Dr. Nedim KOŞUM, Faculty of Agriculture, Ege University, Turkey, the taxonomic position of these plant samples were identified and authenticated. This study complies with relevant institutional, national, and international guidelines and legislation. Other chemicals used were of analytical grade.

### Synthesis of carbon nanoparticles (CNPs)

Olive oil was placed in 100 mL of earthenware pot and cotton wick was used to ignite the fire, which resulted in powder being emitted from the smoke. The powder was collected from the bottom of the earthenware pot using a standardized blade after 5 h and 10 g of the obtained powder was suspended in 200 mL of nitric acid in a 500 mL round bottom flask for refluxing for 28 h. The yellow supernatant was transferred to a conical flask and excess nitric acid was removed from the supernatant using a 3:2 ratio of acetone with water. The contents were then centrifuged at 12,000 rpm for 30 min to extract CNPs, which were then stored for further use^17^.

### Extraction of tea leaf extract (TLE)

A normal hot water extraction process was used to extract the green tea leaves. One hundred milliliters of sterile boiling water was heated to 100 °C and the tea leaves were immersed in it and allowed to seep for 5 min. The TLEs were removed after 5 min and the extract was kept for further use. The green tea leaf extract was centrifuged at 5000 rpm for 5 min. TLE from microsized green tea extract brown colour particles was subjected to FTIR, SEM & EDX.

### Fabrication of nanofibrous airfilter

PVA (8 w/w %) was dissolved in deionized water at room temperature. CNPs of 0.1% and TLE of 1 mL were added to the PVA solution and agitated at 80 °C until an electrospinning homogeneous solution was obtained. To obtain nanofibers, the electrospinning solution used a laboratory electrospinning machine^18^. A DC power supply (Spellman SL150), a syringe pump (NE1000 New Era Pump Systems, Inc) and a metal surfaced drum collector make up the installation. The electrospinning settings were 22 kV applied voltage at 0.15 mL/h flow rate and a needle (23 gauge) tip to drum distance of 20 cm. Finally, the nanofiber peal was obtained from the depositor collector. All experiments were conducted at room temperature. Two other nanofibrous airfilters were electrospun as PVA and PVA: CNPs.

### Functionalization of nanofibrous airfilter (FNA)

Nanofibrous mat specimens measuring 8.0 cm in length, 8.0 cm in width and 0.5 mm in thickness were examined prior to the stabilization process. The prepared cut samples were placed in a muffle furnace, preheated to 280 °C with a heating rate of 1 K/min, and then isothermally treated for 1 h at this temperature. These samples were provided a typical stabilization temperature of 600 °C for 1 h in a muffle furnace, and then allowed to cool before use.

### Nanofiber characterization

Fourier transform infrared (FTIR) spectroscopy measurements were performed using a Nicolet 360 instrument. TGA was used on a thermogravimetric analyser with a high resolution of 2950 TGA (TA, Instru.) Scanning electron microscopy (SEM) images were taken by a 15 kV accelerating voltage with a resolution of 5 nm (Thermo Scientific Apreo S) and using a magnification of 5000 ×. To calibrate the binding energy to C1 *s*, O1 *s* and N *s*, X-ray photoelectron spectroscopy (XPS) analysis was performed with a PHI 5000 Versa Probe-Scanning ESCA Microprobe. Mechanical properties were measured using INSTRON (model 1405) at an extension rate of 5 mm/min. The pore size and distribution of the elecrospun airfilter membrane was determined using a capillary flow porometer based on capllary flow analysis method. The filter resistance was measured with the combination of a flow meter and two electronic pressure transducers that detected the pressure drop through the filtration medium under testing.

### Ultraviolet protective test

UV–Vis spectroscopy was used to assess UV absorption. In one cuboid, the sample is inserted and the reference solvent is placed in the other cuboid. After interacting with UV–Vis light at room temperature, the sample spectra were obtained. All of the tests used a wavelength range of 250–800 nm.

### Antimicrobial test

FNA was evaluated for antimicrobial performance against *Staphylococcus aureus* CECT240 (ATCC 6538p) and *Escherichia coli* ECT434 (ATCC 25922) strains procured from the Turkish type culture collection. Bacterial cultures were subcultured on nutrient agar medium and kept at room temperature (30 ± 2 °C). The antimicrobial performance of FNA was assessed using Ul-Islam et al.^19^. The inoculated samples were placed in bottles and incubated for 24 h at 24 °C with the pH of the agar media for microbial culture kept at 7.0 and the relative humidity at 95%. PVA, PVA: CNPs and PVA: CNPs: TLE samples with a size of 1 × 1 cm were prepared and subjected to the disc diffusion method. After 2 h of UV sterilization, the samples were placed on *E. coli* and *S. aureus* plates. The plates were incubated for 24 h at 37 °C. The diameter of the zone of inhibition against the organism was used to assess antibacterial efficacy.

### Particulate matter (PM_2.5_ & PM_10–2.5_) efficiency of FNA

**Figure 1:**
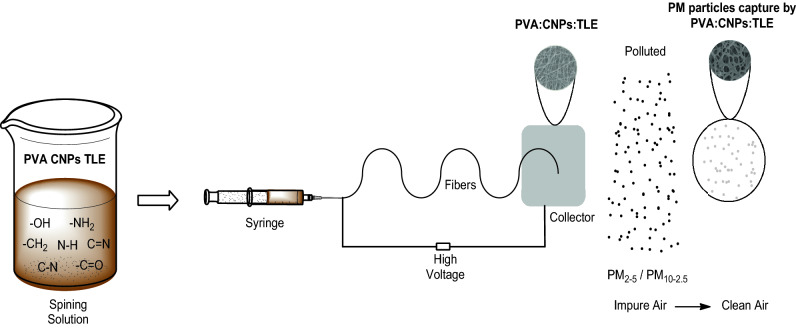
Preparation of FNA-schematic illustration.

The PM removal efficiencies of PVA, PVA: CNPs and PVA: CNPs: TLE were determined in a small scale desigator setup to measure the effect of particle loading on the PM_2.5_ and PM_10–2.5_ removal efficiency of FNA. The experimental setup for assessing the PM_2.5_ & PM_10–2.5_ removal effectiveness as a function is shown in Fig. 1. The efficacy test setup was made of glass plates with dimensions of 750 mm × 60 mm × 60 mm (length × width × height). The middle of the layer was sealed with FNA with dimensions of 60 mm × 60 mm. Air pollution of cigarette smoke was employed to deliver air across the filter and PM_2.5_ and PM_10–2.5_ monitors were tested. Air quality measurements (Testo, 0563 4405, USA) were mounted at the smoking interior and outdoor setup to measure the air quality readings. The temperature and relative humidity in the lab were kept at 25 °C and 60%, respectively, during the experiments.

### Statistical analysis

The results are presented as the mean ± standard deviation (SD) for three individual experiments (n = 3). ANOVA (analysis of variance) and Duncan’s multiple range analysis were performed to determine the significant differences among the different groups. P values of < 0.05 were considered significant.

## Results and discussion

The FNA preparation is depicted in the schematic diagram (Fig. 1).

### Characterization of CNPs and TLE

**Figure 2:**
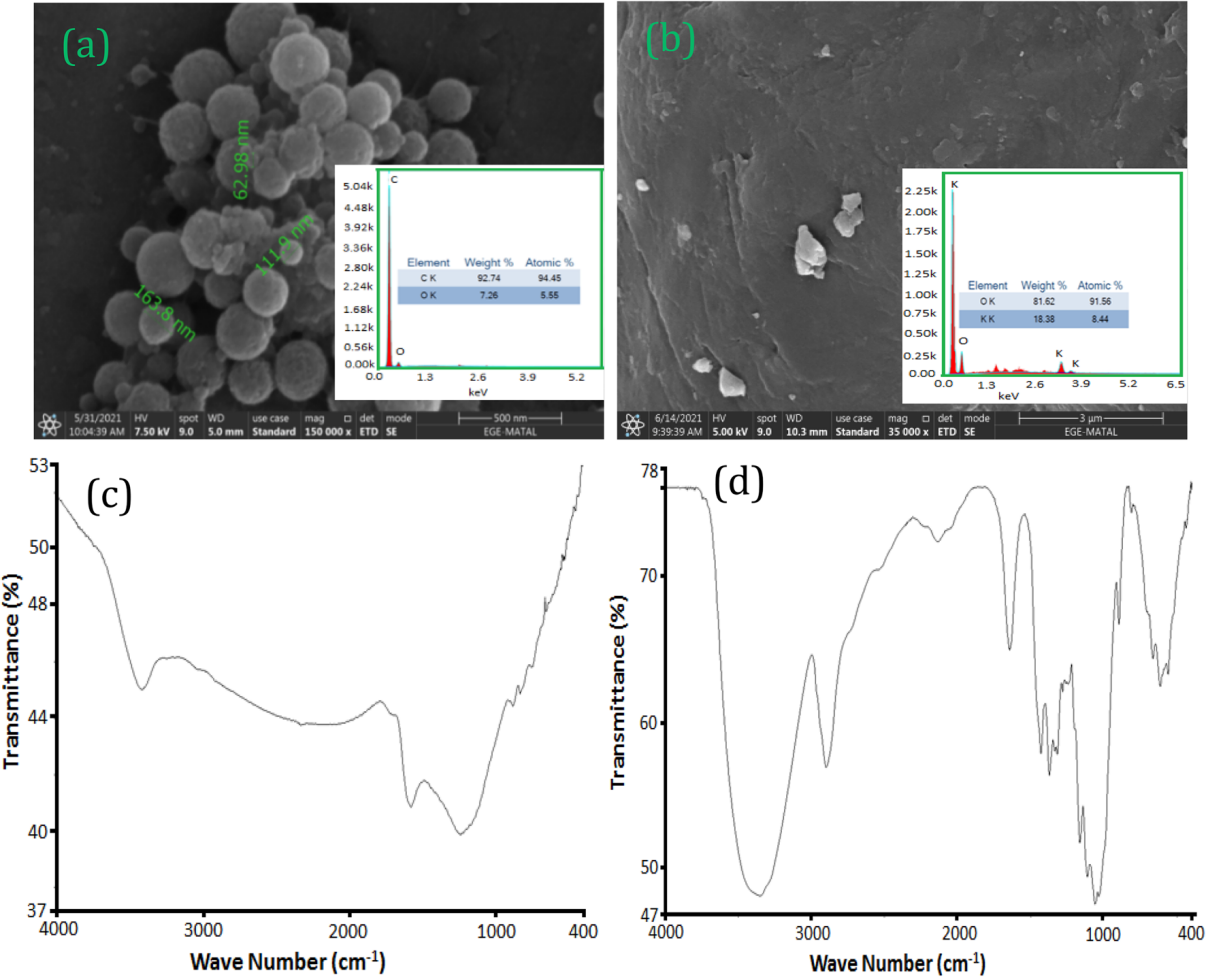
(**a**) SEM of CNPs, (**b**) SEM of TLE, (**c**) FTIR of CNPs, (**d**) FTIR of TLE.

The nanostructural morphology of CNPs and TLE were SEM images illustrated in Fig. 2a and b. CNP spherical particles ranged in size from 20 to 100 nm in diameter with an average particle size of approximately 60 nm. EDX spectrum of CNPs which indicated the presence of pure carbon. The morphology of TLE was smooth and distinct shapes were observed. The EDX spectrum of TLE was found to be free of any contaminants. The FTIR spectra of CNPs (Fig. 2c) exhibited peaks at 1140 cm^−1^ and 859 cm^−1^ representing C–O stretching and C–C stretching groups of CNPs. The TLE (Fig. 2d) peak at 3405 cm^−1^ was due to the N–H and O–H stretching modes of polyphenols^20^.

### Characterization of FNA

#### FTIR

**Figure 3:**
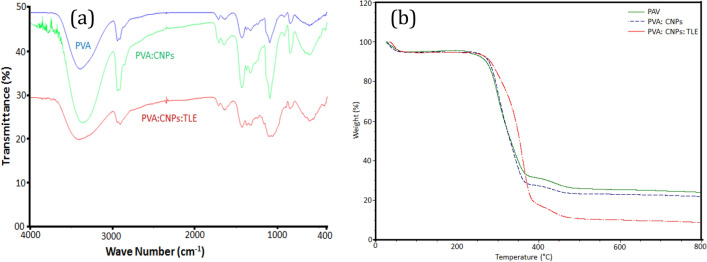
(**a**) FTIR spectra of PVA, PVA: CNPs and PVA: CNPs: TLE and (**b**) TGA spectra of PVA, PVA: CNPs and PVA: CNPs: TLE.

FTIR absorption spectra (Fig. 3a) of PVA, PVA: CNPs and PVA: CNPs: TLE depict the function of wavenumber in the 400–4000 cm^−1^ band. PVA had a distinctive peak in the region of 3000–3500 cm^−1^, which was attributed to intramolecular and intermolecular hydrogen bond O–H stretching vibrations. The crystalline structure of PVA is responsible for the peak at 1141 cm^−1^. The absorption peak at 3380 cm^−1^ assigned to OH stretching of CNPs revealed the presence of hydrocarbons and oxygen in PVA/CNPs electrospun nanofibrous airfilter. The symmetric C–C stretching mode or stretching vibration of the C–O of a segment of the chain where an intramolecular hydrogen bond is generated between two nearby OH groups on the same side of the carbon chain plane is related to this peak^21^. PVA: CNPs: TLE was clearly visible in the region 1500–1800 cm^−1^ which corresponds to the benzene ring in aromatic compounds or pyridine derivatives. The impact of TLE was used to estimate structural changes in PVA/CNPs blend and caused a change in the potential energy distribution along the polymeric chain^22^.

#### TGA

Figure 3a and b shows the TGA curves of PVA, PVA: CNPs and PVA: CNPs: TLE electrospun nanofibrous airfilter. The first stage was weight reduction to remove hydrogen bound water molecules from the polyphenolic structure at temperatures ranging from 100 to 200 °C. The second mass loss that occurred between 200 and 350 °C is linked to the backbone disintegration of PVA and TLE. PVA alone decomposes of side chain breaking at approximately 290 °C. The breakdown of CNPs causes the greatest weight loss in the temperature range of 320 to 480 °C. The OH and COOH groups in the polymeric chains allow the CNPs to interact with TLE and PVA. These interactions might result in the formation of weak intermolecular cross-links between polymeric chains^23^. Thermal deterioration of mineral byproducts causes the final stage which occurs at temperatures exceeding 500 °C.

#### SEM

**Figure 4:**
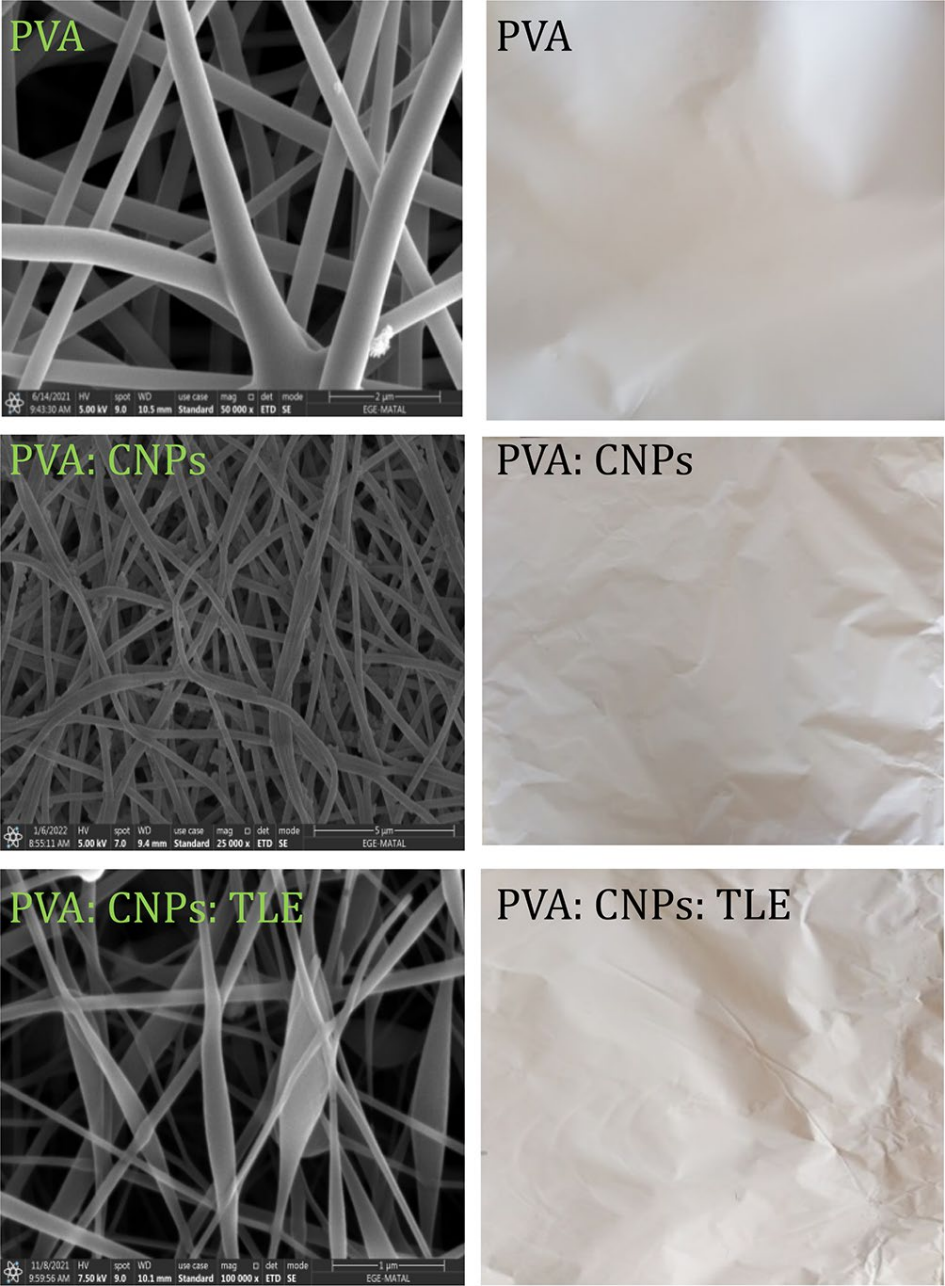
SEM and raw scaffolds of PVA, PVA: CNPs, PVA: CNPs: TLE.

Figure 4 shows SEM images of an PVA, PVA: CNPs and PVA: CNPs: TLE, which were used to evaluate the fiber diameter. PVA, PVA: CNPs and PVA: CNPs: TLE had fiber diameters of 64 ± 15, 83 ± 7 and 123 ± 14 nm, respectively. The combination of PVA, CNPs and TLE in FNA aids in the increase of membrane thickness from 0.2 to 0.4 mm. The PVA had a different morphology, with nanofibers of very larger diameter as compared with the other two samples. It is evident that adding CNPs and TLE to the PM_2.5_ and PM_10-2.5_ removal processes improves the efficiency. The functionalized specimens of PVA, PVA: CNPs and PVA: CNPs: TLE had a smooth texture and were fibrous in nature.

#### Mechanical properties

Mechanical characteristics of airfilter membrane play a important role in determining their end use application perspectives. Mechanical characteristics such as tensile strength, elongation at break, flexing index, water absorption and water desorption were investigated (Table 1). The results reveal that the mechanical properties of PVA: CNPs: TLE were higher than those of the other two samples^24^. This enhanced strength may be due to the impregnation of CNPs and TLE in PVA. Support of CNPs has enhanced the mechanical properties of the airfilter membrane, because these nanoparticles provide reinforcement effect with a combination of tensile strength and flexibility. Roohani-Esfahani et al.^25^ reported that the dispersion microstructure of CNPs play a major role in the reinforcement of electrospun scaffold. The network structure and the interface PVA polymeric layer, surrounding nanoparticles improve the moderate strength and results in increase of mechanical properties.

**Table 1:** Mechanical properties of PVA, PVA: CNPs, and PVA: CNPs: TLE.

Samples	Tensile strength (MPa)	Elongation at break (%)	Flexing index (%)	Water absorption (%)	Water desorption (%)
PVA	15.40 ± 0.18	16.52 ± 0.26	5.52 ± 0.35*	32.66 ± 0.65	40.21 ± 0.01*
PVA: CNPs	17.23 ± 0.08*	18.32 ± 0.09	6.15 ± 0.07	34.48 ± 0.37*	42.16 ± 0.03*
PVA: CNPs: TLE	18.31 ± 0.09	18.95 ± 0.03	6.95 ± 0.03*	35.44 ± 0.41	43.12 ± 0.04

The data are presented as the mean ± SD of three individual experiments.

*p < 0.05. Compared to PVA, using Duncan’s multiple range analysis.

**Figure 5:**
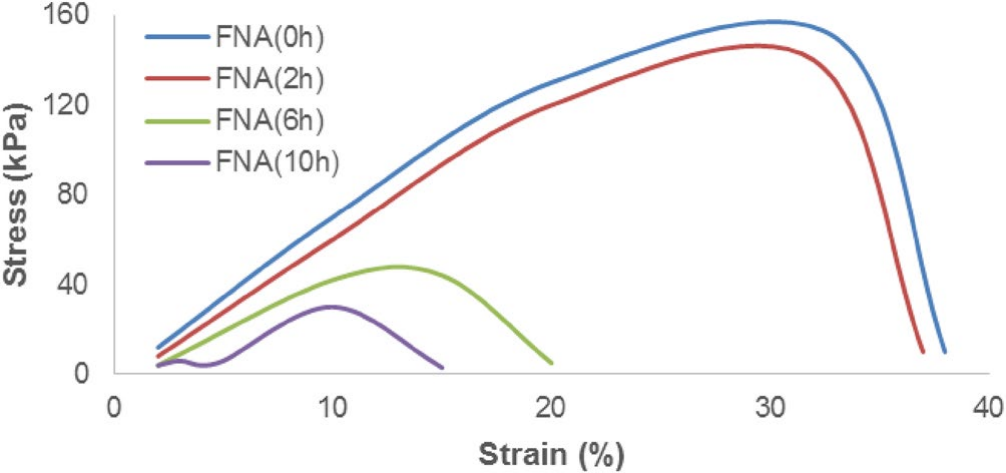
Stress–strain curves of the FNA.

Water absorption and desorption play an important role in scaffold properties and their dimensional stability. Water absorption and desorption significantly (p < 0.05) increased in PVA: CNPs: TLE compared to the other two samples. These enhanced water absorption properties of electrospun scaffold could be attributed to the tea leaf extract and the formation of hydrogen bond between TLE and PVA^26^. Most of all leaf extract are hydrophilic in nature with a moisture content of 6–10% due to the presence of cellulose in cell membrane^27^. Water absorption and desorption capacity of a electrospun membrane play a considerable role in choosing its use in airfilter. Since, maintaining a dry product surface is essential to prevent moisture content and microbial growth. The durability test of FNA is given in Fig. 5. The stress–strain results reveal that FNA (after 10 h) had higher durability than the other h.

#### Pore size and pressure drop analysis

**Figure 6:**
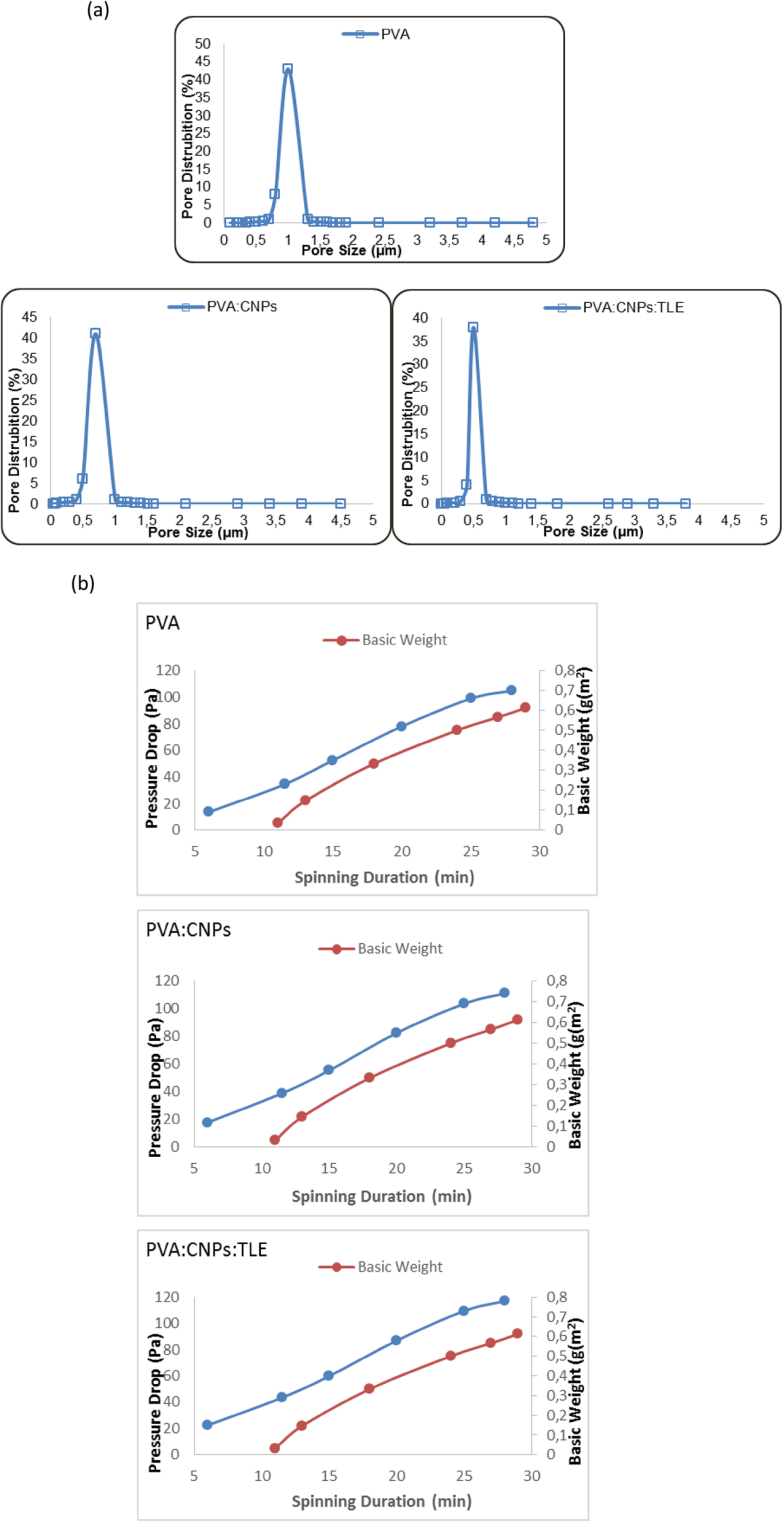
(**a**) Pore size distribution curve of PVA, PVA: CNPs and PVA: CNPs: TLE, (**b**) Pressure drop of PVA, PVA: CNPs and PVA: CNPs: TLE.

Figure 6a showed the pressure drop of PVA, PVA: CNPs and PVA: CNPs: TLE. The pressure drop value of PVA, PVA: CNPs and PVA: CNPs: TLE are 98, 105 and 110 Pa, respectively. The pore size distripution for PVA, PVA: CNPs and PVA: CNPs: TLE obtained from different concentration were shown in Fig. 6b. The pore size distribution of PVA: CNPs: TLE is mainly determined by the morphology and size of the nanofibers. 5.24 m^2^/g surface area, 0.15 cm^3^/g total pore volume and 76.90 nm average pore width was observed in PVA: CNPs: TLE. The surface to volume area, narrow distribution and small pore size, as well as the significantly high porosity, enable electrospun membranes to efficiently separate contaminants in air treatment^28^. The results demonstrate that PVA: CNPs: TLE had better pore size compared to PVA and PVA: CNPs.

#### Antimicrobial activity

**Figure 7:**
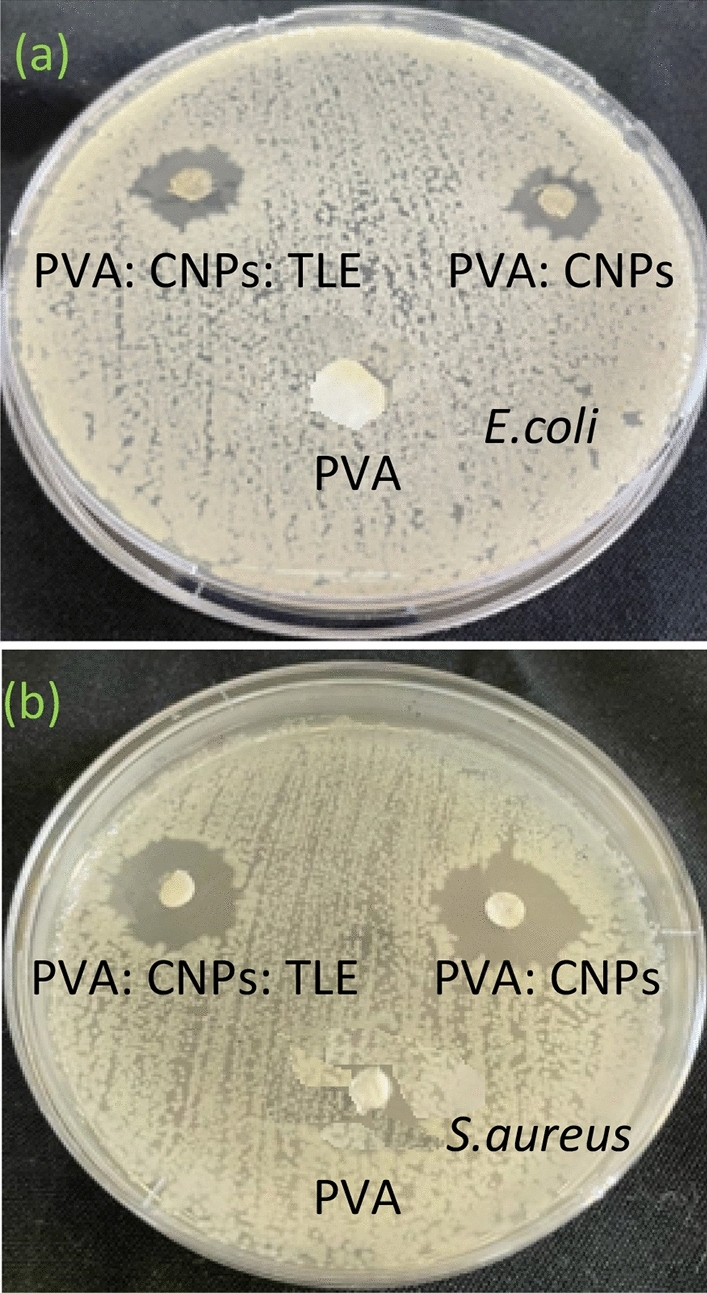
(**a**) Inhibition zone of PVA, PVA: CNPs, PVA: CNPs: TLE against *E. coli*, (**b**) Inhibition zone of PVA, PVA: CNPs, PVA: CNPs against *S. aureus.*

The antimicrobial properties of PVA, PVA: CNPs and PVA: CNPs: TLE were tested following the disc diffusion method against *E. coli* and *S. aureus*. The results obtained from this clearly mention the inhibition zone of PVA, PVA: CNPs and PVA: CNPs: TLE (Fig. 7a,b). A shown in Table 2, PVA: CNPs: TLE had exhibited antimicrobial activity against the *E.coli* and *S. aureus*. Brady-Estevez^29^ have reported that membrane airfilter form containing carbon nanoparticles played an important role in enhancing the antimicrobial activity against gram (−) and gram (+) bacteria. Microbial growth can be suppressed by the entry of CNPs into the cell membrane and also hindered by the formation of reactive oxygen species^30^. The antibacterial activity of tea leaf extract is attributed to polyphenols, which are two benzene rings as A^-^and B^-^ and pyridine derivatives are well characterized^31^. The physical interaction between the microbial membrane and fullerenes, in which CNPs cause DNA fragmentation in the cell membrane due to the particles’ high surface hydrophobicity^32^.

**Table 2:** Antimicrobial activity of PVA, PVA: CNPs, PVA: CNPs: TLE.

Samples	Zone of inhibition (mm)	
	*E. coli*	*S. aureus*
PVA	0.00 ± 0.00	0.00 ± 0.00
PVA: CNPs	3.12 ± 0.02	3.32 ± 0.10
PVA: CNPs: TLE	4.10 ± 0.00	4.13 ± 0.05

The data are presented as the mean ± SD of three individual experiments.

#### Ultraviolet protective test

**Figure 8:**
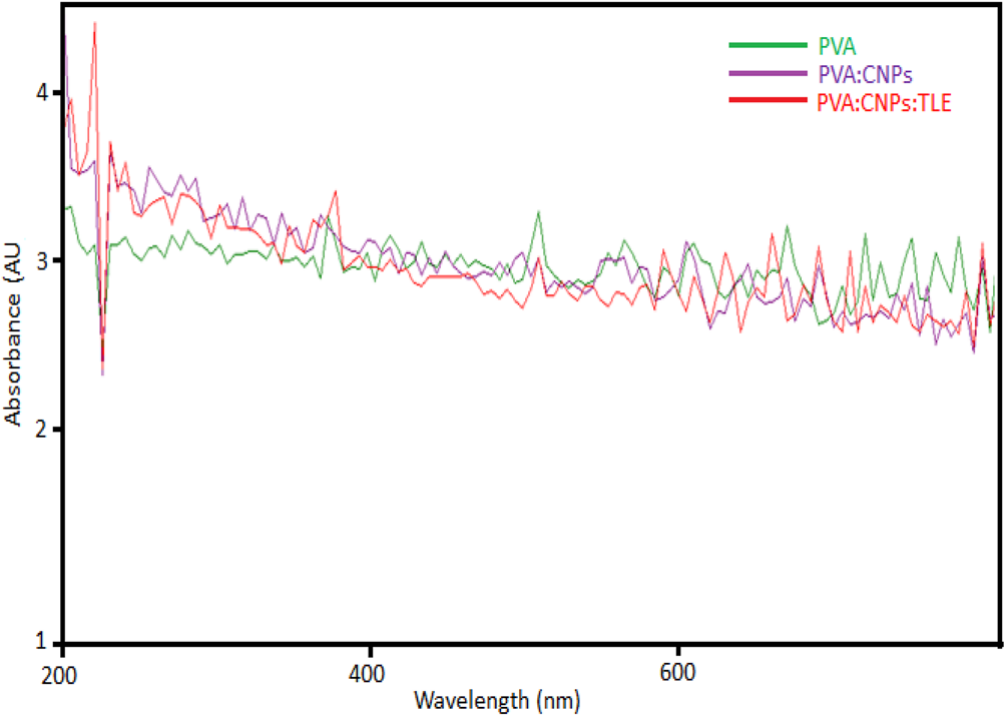
UV absorption spectrum of PVA, PVA: CNPs and PVA: CNPs: TLE.

Figure 8 shows the UV spectra of PVA, PVA: CNPs and PVA: CNPs: TLE with nanofiber diameters of 175, 98 and 95 nm. According to the results, as the nanofiber diameter decreases from 175 to 95 nm. The UV-production performance of electrospun PVA: CNPs: TLE nanofibrous mats was determined by the nanofiber diameter according to our research observations. Smaller pore sizes have been demonstrated to produce better UV-production characteristics because they absorb UV radiation more effective^33^.

#### Particulate matter (PM_2.5_ & PM_10–2.5_) efficiency of FNA

**Figure 9:**
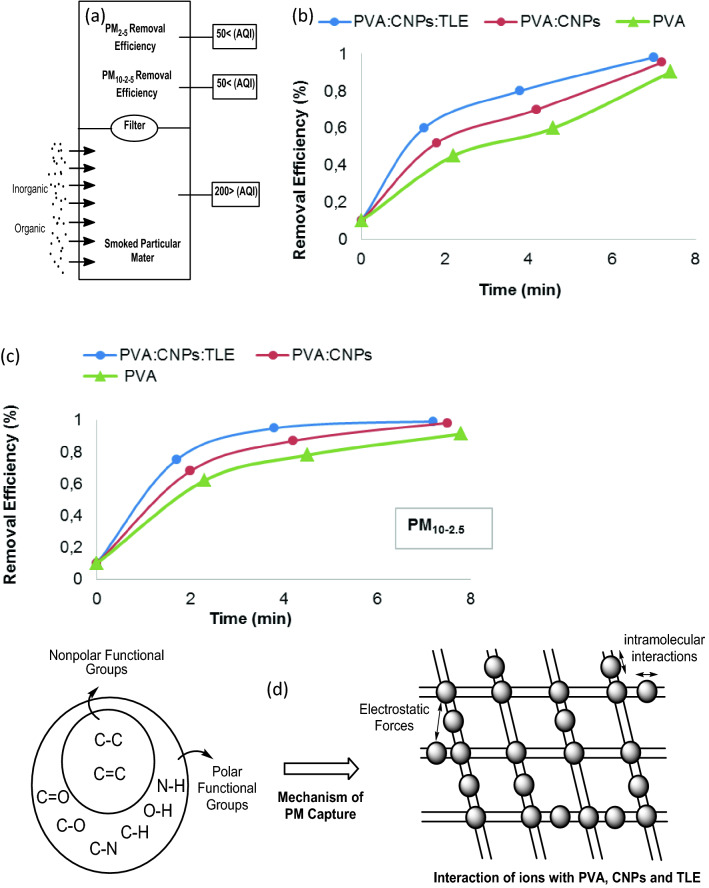
(**a**) The setup designed for testing the efficiency capture of PM_2.5_ and PM_10–2.5_ capture (**b**) PM_2.5_ capture efficiency of PVA, PVA: CNPs and PVA: CNPs: TLE (**c**) PM_10–2.5_ capture efficiency of PVA, PVA: CNPs and PVA: CNPs: TLE (**d**) Schematic showing the mechanism of PM capture.

Figure 9a depicts the test equipment used in this study to remove PM particles from the chamber which includes an our FNA (PVA, PVA: CNPs and PVA: CNPs: TLE). Figure 9b and c shows the PVA: CNPs: TLE, PM_2.5_ removal efficiency of 99.21% and PM_10–2.5_ efficiency of 99.28%. PVA: CNPs showed PM_2.5_ and PM_10–2.5_ removal efficiencies of 95.31% and 98.34%, respectively. PVA had a PM_2.5_ removal efficiency of 90.23% and 91.45% PM_10–2.5_ removal efficiency. Hence, PVA: CNPs: TLE had the best performance as a smoke airfilter. The filter particle counter and removal efficiency give a PM_2.5_ and PM_10–2.5_ calculated results according to a previous study in the literature^34^.

The PM capture process and demonstration are shown in Fig. 9d. PM particles captured by the nanofibers were bound tightly on the surface. In the case of PM, numerous mechanisms, such as diffusion, interception, inertia and gravity, work together to capture these particles^35^. The air filtration process was dominated by interception for particles with a diameter higher than the pore size of the filters. Gravity plays a crucial role in particle capture with the airflow perpendicular to the ground^36^. On the outside of PM particles, there are many functional groups with high polarity such as C–O, –SO_3_H, C–N and –NO_3_. As a result, functional groups as well as materials that were easily surface modified showed tremendous promise for proactive capture. Hui et al.^37^ examined the properties, composition, morphology and capture mechanism of PM particles using SEM, EDX and XPS.

**Figure 10:**
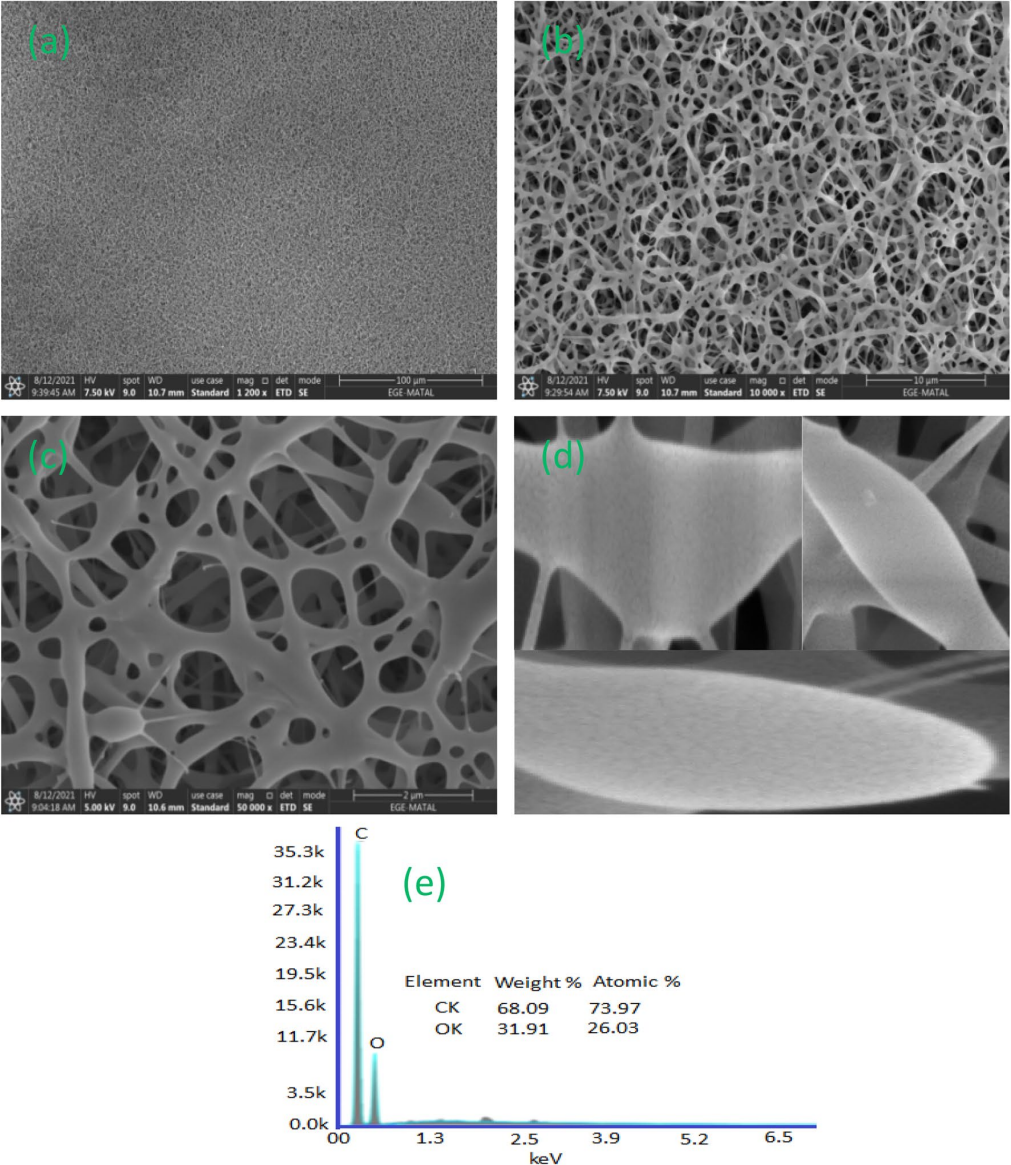
(**a**–**c**) SEM images of PVA, PVA: CNPs, PVA: CNPs: TLE after PM capture experiment, (**d**) SEM shows PM particles formed around the PVA: PVA: CNPs: TLE nanofilter, (**e**) EDX of PVA: PVA: CNPs: TLE after PM capture.

After the PM_2.5_ and PM_10–2.5_ removal efficiency tests, the FNA was SEM images are shown in Fig. 10a–c. As a result, the removal effectiveness of PM_2.5_ and PM_10–2.5_ was significantly improved as PM particles firmly wrapped around the nanofibers. The size distribution of smoke PM particles ranges from 400 nm, with the majority of particles being 1 mm. By decreasing the fiber diameter in the range of 100 to 200 nm, the PM_2.5_ capture efficiencies increased. The fibrous structure of the electrospun scaffold and spherical shape were clearly observed in the results. Figure 10d shows the airfilter operation and tight binding of PM particles to produce an outstanding capture performance of the electrospun scaffold which was validated by microscopy. These results showed that the fiber diameter of PVA: CNPs: TLE was less than 150 nm, indicating that it has the ability to capture PM_2.5_ and PM_10–2.5_. Figure 10e shows the elemental analysis of the prepared PVA: CNPs: TLE using EDX. Nanofiber diameters ranging from 60 to 200 nm have been found to improve particle capture of air molecules in previous studies^38^. In general, the pore size of a nanofiber filter has a weak relation with the PM_2.5_ removal efficiency^39^. Nanofibers have proven to be useful in airfilters because of their small diameter and high surface to volume ratio, which improve particle absorption by interception^40^.

**Figure 11:**
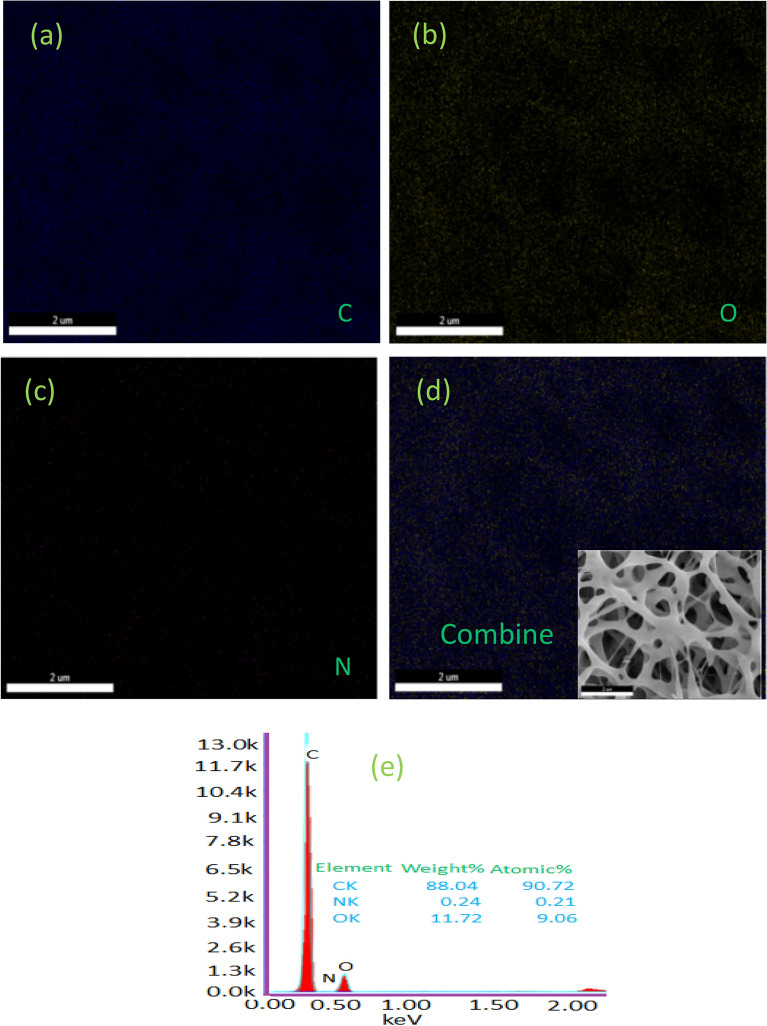
(**a**–**d**) Elemental mapping of PVA: CNPs: TLE after PM capture, (**e**) EDX of PVA: CNPs: TLE after PM capture.

Elemental mapping (Fig. 11a–d) was performed on PVA: CNPs: TLE after exposure to PM_2.5_ and the PM_10–2.5_ removal effect. The surface of the nanofiber was successfully found by the uniform distribution of the elements. The EDX spectra of PM_2.5_ and PM_10–2.5_ removal by PVA: CNPs: TLE are shown in Fig. 11e. Carbon, oxygen and nitrogen element mapping seen on the nanofiber surface.

#### XPS

**Figure 12:**
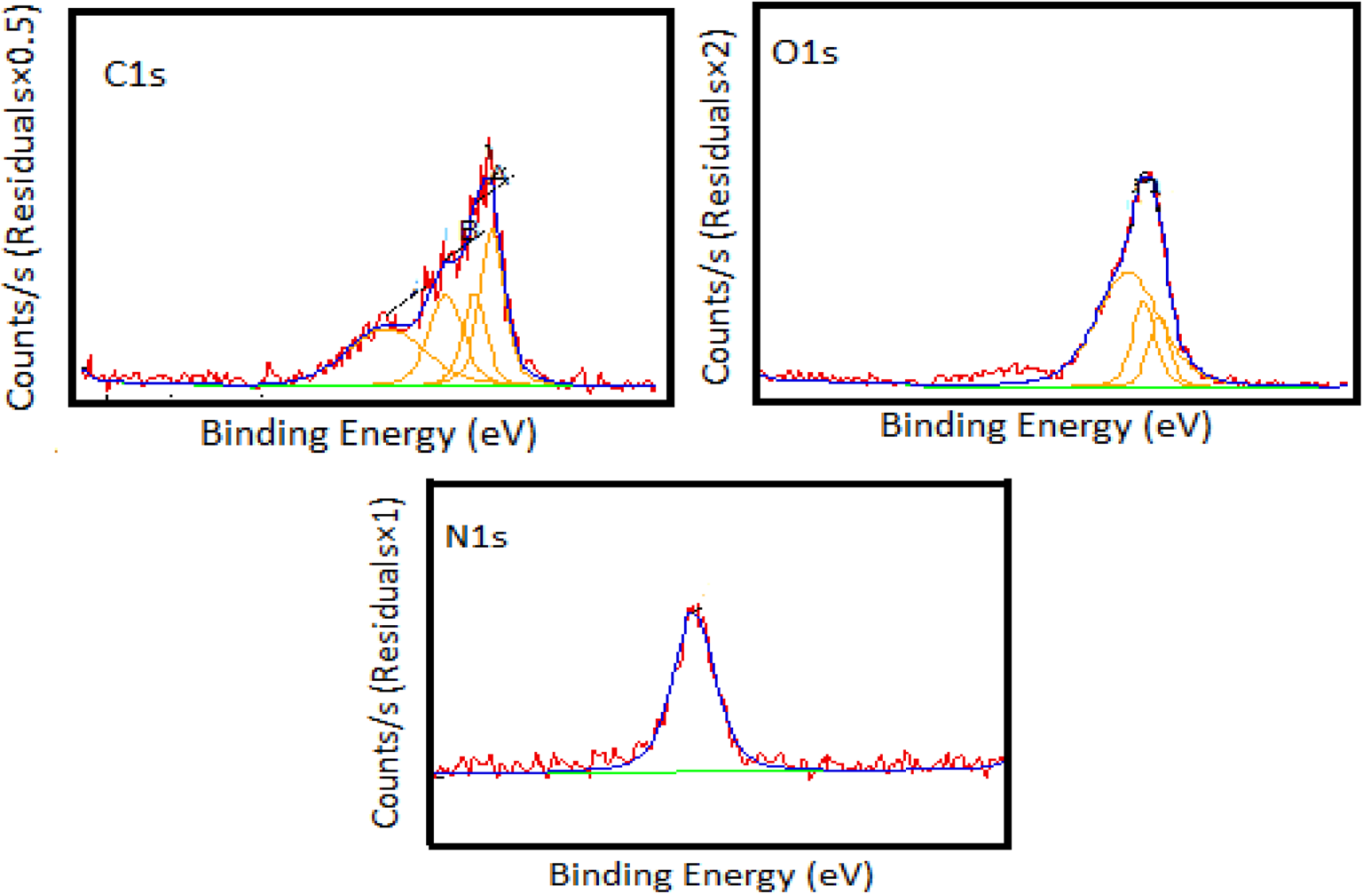
EDX analysis of PVA: CNPs: TLE after PM capture.

The presence of CNPs and TLE in PVA electrospun nanofibers was confirmed using surface chemistry scan spectra. The characterization of PM using X-ray photoelectron spectroscopy (XPS) is shown in Fig. 12. The XPS spectrum shows that the C1 s signal comprises three significant peaks at 284.7, 285.9 and 286.6 eV, which correspond to C–C, C–O and C=O bonds. The O1 *s* peaks showed the presence of C-O and C=O at 533.1 and 531.9 eV, respectively. In addition, a minor amount of N1 *s* was present on the surface of smoke particles, which was shown at the peak of 400.8 eV. The overall results was confirmed that C, O and N are three elements on the contaminated air PM surface and that the PM surface contained 58.5% carbon, 36.1% oxygen and 5.4% nitrogen, respectively. Chong et al.^41^ reported that three elements had PM_2.5_ capture surfaces.

In this research, PM was created by smoke burning. Exhaust smoke polluting gases such as SO2, NO2, CO2, CO and volatile organic compounds including polycyclic aromatic hydrocarbons, xylenes, benzene, toluene and aldehydes contain 40 mg g^−1^^33^. Electrospun nanofibrous membranes capture dust particles on their surfaces, which can be easily removed by back flushing or other mechanical methods^42^. In summary, the results of airfilter efficiency analysis suggested that the PVA: CNPs: TLE nanoairfilter promoted the process of PM_2.5_ and PM_2.5–10_ capture, indicating their great potential as airfilter applications.

## Conclusion

In this research, we designed and developed electrospun FNA for PM_2.5_ and PM_2.5–10_ particle capture materials. Electrospun FNA exhibited physicochemical, mechanical and antimicrobial activity. An efficiency test demonstrated that particulate matter (PM_2.5_ & PM_10–2,5_) capture was significantly PM removed by these polluted air samples. All results show that these PVA: CNPs: TLE nanofilters have excellent PM filtration properties compared to PVA: CNPs and PVA filters. The prepared air filter was able to efficiently remove PM_2.5_ and PM_2.5–10_ from polluted air thus proving to be a viable and cost-effective strategy. In summary, these unique PVA: CNP: TLE nanofilters can be widely used in commercial, domestic and industrial places.

## References

[1] Jaison J, Ahmed B, Yen SC, Alain D, Michael KD (2018). Review on nanoparticles and nanostructured materials: History, sources, toxicity and regulations. Beilstein J. Nanotechnol..

[2] Huang X, Jiao T, Liu Q (2019). Hierarchical electrospun nanofibers treated by solvent vapor annealing as air filtration mat for high-efficiency PM2.5 capture. Sci. China. Mater..

[3] Yang X, Zhang L, Chen X, Liu F, Shan A, Liang F, Li X, Wu H, Yan M, Ma Z (2021). Long-term exposure to ambient PM2.5 and stroke mortality among urban residents in northern China. Ecotoxicol. Environ. Saf..

[4] Brauer M, Casadei B, Harrington RA, Kovacs R, Sliwa K (2021). Taking a stand against air pollution—the impact on cardiovascular disease: A Joint Opinion from the World Heart Federation, American College of Cardiology, American Heart Association, and the European Society of Cardiology. J. Am. Coll. Cardiol..

[5] Monda S (2021). Nanomaterials for UV protective textiles. J. Ind. Text..

[6] Platt SM, Haddad IE, Pieber SM, Huang RJ, Zardini AA, Clairotte M, Suarez-Bertoa R, Barmet P, Pfaffenberger L, Wolf R, Slowik JG, Fuller SJ, Kalberer M, Chirico R, Dommen J, Astorga C, Zimmermann R, Marchand N, Hellebust S, Temime-Roussel B, Baltensperger U, Prévôt ASH (2014). Two-stroke scooters are a dominant source of air pollution in many cities. Nat. Commun..

[7] Khalid B, Bai X, Wei H, Huang Y, Wu H, Cui Y (2017). Direct blow-spinning of nanofibers on a window screen for highly efficient PM25 removal. Nano. Lett..

[8] Sundarrajan S, Tan KL, Lim SH (2014). Electrospun nanofibers for air filtration applications. Procedia Eng..

[9] Hutten IM (2007). Handbook of Nonwoven Filter Media.

[10] Zhu C, Dobryden I, Rydén J, Öberg S, Holmgren A, Mathew AP (2015). Adsorption behavior of cellulose and its derivatives toward Ag(I) in aqueous medium: An AFM, spectroscopic, and DFT study. Langmuir.

[11] Rios JL, Recio MC (2005). Medicinal plants and antimicrobial activity. J. Ethnopharmacol..

[12] Sundarrajan S, Ramakrishna S (2013). New directions in nanofiltration applications: Are nanofibers the right materials as membranes in desalination?. Desalination.

[13] Zhang L, Li L, Wang L, Nie J, Ma G (2020). Multilayer electrospun nanofibrous membranes with antibacterial property for air filtration. Appl. Surf. Sci..

[14] Tasis D, Tagmatarchis N, Bianco A, Prato M (2006). Chemistry of carbon nanotubes. Chem. Rev..

[15] Ma PC, Siddiqui NA, Marom G, Kim JK (2010). Dispersion and functionalization of carbon nanotubes for polymer-based nanocomposites: A review. Compos. A Appl. Sci. Manuf..

[16] Shenoy SL, Bates WD, Frisch HL, Wnek GE (2005). Role of chain entanglements on fiber formation during electrospinning of polymer solutions: Good solvent, non-specific polymer-polymer interaction limit. Polymer.

[17] Dubey P, Muthukumaran D, Dashrupa S, Ruba M, Sabyasachi S (2005). Synthesis and characterization of water-soluble carbon nanotubes from mustard soot. Pramana. J. Phys..

[18] Senthil R, Berly R, Bhargavi T, Gobi N (2018). Electrospun PVA/collagen nanofiber scaffold hybridized by graphing oxide by accelerated wound healing. Int. J. Artif. Organ..

[19] Ul-Islam M, Khan T, Khattak WA, Park JK (2013). Bacterial cellulose-MMTs nanoreinforced composite films: Novel wound dressing material with antibacterial properties. Cellulose.

[20] Loo YY, Chieng BW, Nishibuchi M, Radu S (2012). Synthesis of silver nanoparticles by using tea leaf extract from *Camellia sinensis*. Int. J. Nanomed..

[21] Mansur HS, Sadahira CM, Souza AN, Mansur AAP (2008). FTIR spectroscopy characterization of poly(vinyl alcohol) hydrogel with different hydrolysis degree and chemically crosslinked with glutaraldehyde. Mater. Sci. Eng. C..

[22] Tawansi A, Zidan H, Moustafa Y, Eldumiaty A (1997). Optical and electrical properties of NiCl_2_ filled PVC films. Phys. Scr..

[23] Zhou J, Shaoting L, Hongxia Z, Ji L, Buxuan L, Yanfei X, Xuanhe Z, Gang C (2020). Dynamic intermolecular interactions through hydrogen bonding of water promote heat conduction in hydrogels. Mater. Horizon.

[24] Senthil R, Basaran B, Vijayan S, Mert A, Bayraktar O, Wilson Aruni A (2020). Electrospun nano-bio membrane for bone tissue engineering application: A new approach. Mater. Chem Phys..

[25] Roohani-Esfahani SI, Nouri-Khorasani S, Lu ZF, Appleyard RC, Zreigat H (2011). Effect of bioactive glass nanoparticles on the mechanical and biological behaviour of composite coated scaffolds. Acta Biomater..

[26] Senthil R, Serdar Batıkan K, Hemalatha T, Wilson Aruni A, Sendemir A, Cem T (2022). Cellulose based electrospun nanofilters: Perspectives on tannery effluent waste water treatment. Cellulose.

[27] Justiz-Smith NG, Virgo GJ, Buchanan VE (2008). Potential of Jamaican banana, coir, bagasse fiber as composite materials. Mater. Charact..

[28] Yu X, Li C, Tian H, Yuan L, Xiang A, Li J, Wang C, Rajulu AV (2020). Hydrophobic cross-linked zein-based nanofibers with efficient air filtration and improved moisture stability. Chem. Eng. J..

[29] Brady-Estevez AS, Kang S, Elimelech M (2008). A single-walled-carbon- nanotube filter for removal of viral and bacterial pathogens. Small.

[30] Kang S, Herzberg M, Rodrigues DF, Elimelech M (2017). Antibacterial effects of carbon nanotubes: Size does matter. Langmuir.

[31] Zaveri NT (2006). Green tea and its polyphenolic catechins: Medicinal uses in cancer and noncancer applications. Life. Sci..

[32] Moor KJ, Osuji CO, Kim JH (2015). Antimicrobial photodynamic therapy with fulleropyrrolidine: Photoinactivation mechanism of *Staphylococcus aureus*, in vitro and in vivo studies. Appl. Microbiol. Biotechnol..

[33] Duleba-Majek M (2009). Transmission of UV radiation through woven fabrics in dependence on the inter-thread spaces. Fibres. Text. East Eur..

[34] Chong L, Po-Chun H, Hyun-Wook L, Meng Y, Guangyuan Z, Nian YC, Chen KP, Yu WC, Shao CH, Tseng WC (2018). Novel mold-resistant building materials impregnated with thermally reduced nano-silver. Indoor Air.

[35] Wang CS, Otani Y (2012). Removal of nanoparticles from gas streams by fibrous filters: A review. Ind. Eng. Chem. Res..

[36] Li P, Wang C, Zhang Y, Wei F (2014). Air filtration in the free molecular flow regime: A review of high-efficiency particulate air filters based on carbon nanotubes. Small.

[37] Hui L, Chunyan C, Jianying H, Zhong C, Guoqiang C, Yuekun L (2020). Progress on particulate matter filtration technology: Basic concepts, advanced materials, and performances. Nanoscale.

[38] Zhao X, Wang S, Yin X, Yu J, Ding B (2016). Slip-effect functional air filter for efficient purification of PM_2.5_. Sci. Rep..

[39] Wang Z, Zhao C, Pan Z (2015). Porous bead-on-string poly (lactic acid) fibrous membranes for air filtration. J. Colloid Interface Sci..

[40] Leung WWF, Sun Q (2020). Charged PVDF multilayer nanofiber filter in filtering simulated airborne novel coronavirus (COVID-19) using ambient nano-aerosols. Sep. Purif. Technol..

[41] Lin TC, Krishnaswamy G, Chi DS (2008). Incense smoke: Clinical, structural and molecular effects on airway disease. Clin. Mol. Allergy..

[42] Thavasi V, Singh G, Ramakrishna S (2008). Electrospun nanofibers in energy and environmental applications. Energy Environ. Sci..

